# Carcinoma associated fibroblasts small extracellular vesicles with low miR-7641 promotes breast cancer stemness and glycolysis by HIF-1α

**DOI:** 10.1038/s41420-021-00524-x

**Published:** 2021-07-08

**Authors:** Yonglei Liu, Fanli Hua, Yanxia Zhan, Yanfei Yang, Jianfang Xie, Yunfeng Cheng, Feng Li

**Affiliations:** 1 Research Center, Zhongshan Hospital Qingpu Branch, Fudan University, Shanghai, China; 2 Department of Hematology, Zhongshan Hospital Qingpu Branch, Fudan University, Shanghai, China; 3 Research Center, Zhongshan Hospital, Fudan University, Shanghai, China; 4 Department of Hematology, Zhongshan Hospital, Fudan University, Shanghai, China

**Keywords:** Breast cancer, Mechanisms of disease, Non-coding RNAs

## Abstract

Fibroblasts play an important role in cancer development and progression. Small extracellular vesicles (sEVs) are one type of extracellular vesicles, which mediate the interaction between cancer-associated fibroblasts and cancer cells by transferring their contents. However, the roles of sEVs from cancer-associated fibroblasts on breast cancer stem cell properties are largely unraveled. The purpose of this study was to explore the roles of sEVs from cancer-associated fibroblasts on breast cancer progression. The miRNA array data showed a different miRNA profile between CAFs sEVs and normal fibroblasts sEVs. By verification using real-time RT-PCR, the data analysis indicated that miR-7641 levels were lower in sEVs from CAFs compared with NFs. The cellular functions were assayed and the results indicated that CAFs derived sEVs with low miR-7641 levels suppressed breast cancer cell survival, glycolysis, and stem cell properties via the HIF-1α pathway. Collectively, these findings indicated that sEVs from CAFs promoted breast cancer stem cell properties and glycolysis via miR-7641/HIF-1α, which was a possible new way for targeting breast cancer.

## Introduction

Tumor stroma cells, cancer cells, extracellular matrix (ECM), and the secreted molecule or other contents constitute tumor microenvironment. Carcinoma-associated fibroblasts (CAFs) are the main cell type in tumor stroma and the emerging evidence indicates that CAFs play important roles in cancer progression [1, 2]. CAFs are the most prominent cell type in the cancer niche, which induce therapeutic resistance, metastasis, cancer stemness, and metabolism changes. In the tumor tissues, CAFs interact with other cells by direct or indirect interaction [3–5].

The cell-to-cell interaction between cancer cells and stromal cells is accomplished via paracrine mechanism including growth factors, chemokines, and proteases by extracellular vehicles (EVs) [6]. EVs with 30–150 nm diameter are called small extracellular vesicles (sEVs) [4, 6, 7]. The sEVs act as natural vehicles delivering growth factors, chemokines, mRNA, microRNA, and other soluble mediators to recipient cells [7, 8]. The sEVs from tumor stroma fibroblasts affect cancer cell biological functions and signal pathways [8]. The sEVs from stromal cells could be transferred into breast cancer cells, which lead to cancer phenotype changes [9–12]. For example, miR-522 from CAFs suppressed ferroptosis and promoted acquired drug resistance in gastric cancer [13]. CAFs exosomes suppress immune ability in breast Cancer by miR-92/PD-L1 [14]. CAFs exosomal miR-17-5p promoted colorectal cancer aggressive behaviors [15]. Previous reports indicate that CAFs sEVs involve in cancer biological processes. But, the functions and mechanism of sEVs from CAFs are still unclear.

In the present study, the effects of sEVs from the CAFs on the breast cancer cell biological process were investigated. The sEVs from normal fibroblasts (NFs) and CAFs were isolated and used to investigate the cellular functions. MiR-7641 was a down-regulated miRNA in the sEVs from CAFs by real-time RT-PCR verification. The cellular functions including proliferation, metastasis, glycolysis, and stem cell features were assayed and the results indicated that CAFs sEVs with low levels of miR-7641 promoted breast cancer stem cell properties via the HIF-1α pathway.

## Results

### The sEVs from CAFs increased breast cancer cell survival and metastasis ability

**Fig. 1:**
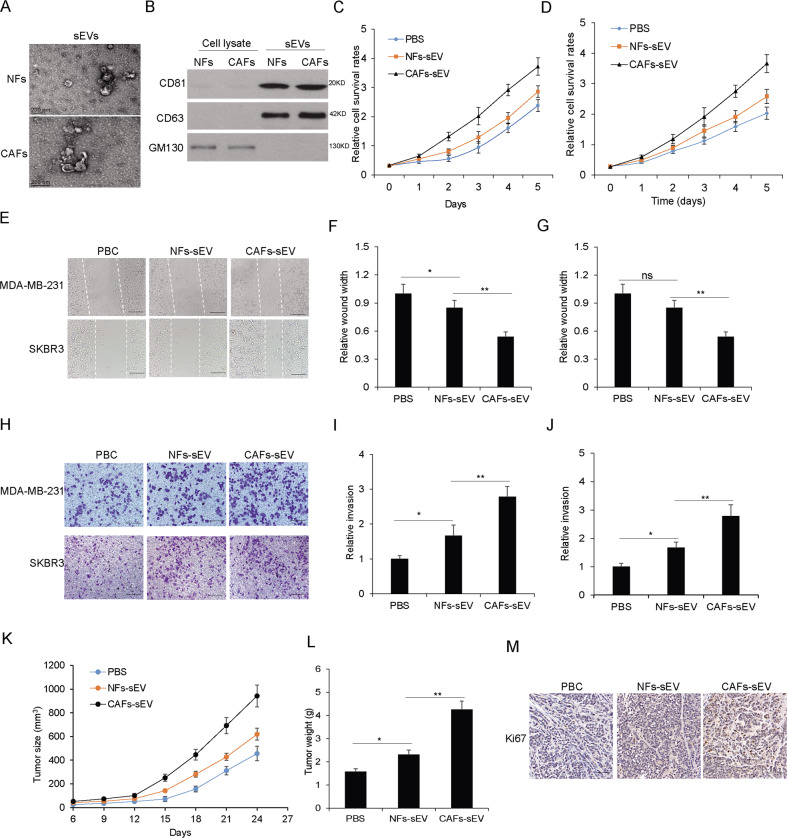
The sEVs from CAFs increased breast cancer cell survival and metastasis ability. **A** The representative picture of sEVs from NFs or CAFs. Transmission electron microscopy was used to take the pictures. **B** The sEVs markers were identified. The total protein was isolated from NFs or CAFs derived sEVs and performed for CD81, CD63, and GM130 immunoblotting by western blotting. **C**, **D** Cell proliferation of MDA-MB-231 and SKBR3 cells exposed to CAFs-sEV and NFs-sEV (25 µg/ml) assayed by CCK8. **E** Wound healing assay was used to examine cell migration of MDA-MB-231 and SKBR3 cells with CAFs-sEV or NFs-sEV treatment (25ug/ml) (scale bars, 100 µm). **F**, **G** Data analysis of (**E**). **H** Transwell system was used to examine cell invasion of MDA-MB-231 and SKBR3 cells with CAFs-sEV or NFs-sEV treatment (25 µg/ml) (scale bars, 100 µm). **I**, **J** Data analysis of (**H**). **K** Tumor growth in vivo. **l** the average tumor weight. **M** IHC straining for Ki67 (scale bars, 50 µm). *p < 0.05, **p < 0.01.

To explore the role of CAFs sEVs on breast cancer cellular biological functions, firstly, sEVs from NFs and CAFs were isolated and taken by transmission electron microscopy (TEM) (Fig. 1A). The sEVs markers like CD63, CD81, and GM130 protein levels were evaluated in sEVs from NFs or CAFs and the cells (Fig. 1B). In order to learn whether CAFs sEVs have an influence on breast cancer cellular functions, SKBR3 and MDA-MB-231 cells were in the presence of CAFs-sEV or NFs-sEV. Cell viability was assayed by CCK8, which showed that CAFs-sEV could enhance the cellular viability of SKBR3 and MDA-MB-231 cells (Fig. 1C–D). To know whether sEVs from the CAFs play the role in migration, SKBR3 and MDA-MB-231 cells were in the presence of CAFs-sEV or NFs-sEV for 48 h and performed for cell migration by wound healing assay, and the result showed that cancer cells with CAFs-sEV treatment had a higher migratory ability (Fig. 1E–G). There were similar results in cell invasion ability using transwell assay (Fig. 1H–J). The in vivo data showed that CAFs-sEV promoted breast cancer growth (Fig. 1K), and tumor weight (Fig. 1L). Ki67 straining showed CAFs-sEV increased tumor growth (Fig. 1M). The data indicated that sEVs from CAFs accelerated breast cancer cell survival and metastasis ability.

### The sEVs from CAFs increased the population of breast cancer stem cells and glycolysis

**Fig. 2:**
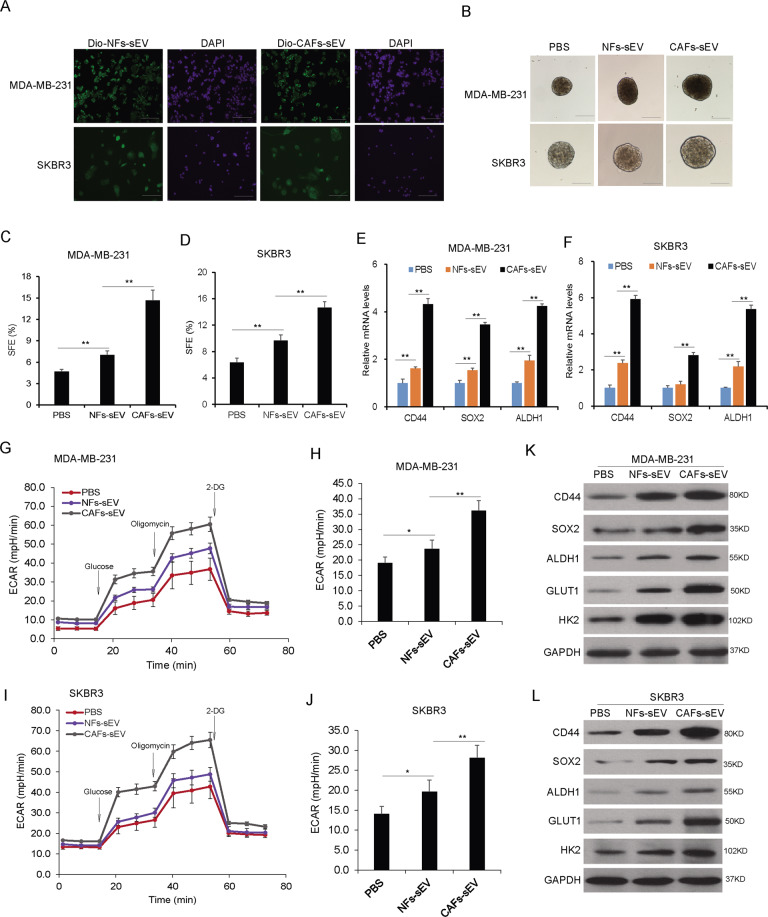
The sEVs from CAFs increased the population of breast cancer stem cells and glycolysis. **A** MDA-MB-231 cells could uptake sEVs from CAFs or NFs. sEVs were labeled with Dio and co-cultured with the cells for 6 h and sEVs were observed under a microscope (scale bars, 100 µm). **B** CAFs derived sEVs increased the population of breast cancer stem cells. MDA-MB-231 and SKBR3 cells were treated with CAFs-sEV or NFs-sEV for sphere formation analysis and the spheres were observed under a microscope (scale bars, 100 µm). **C**, **D** Sphere formation rates of MDA-MB-231 and SKBR3 cells. **E**, **F** The expression of stem cell markers in MDA-MB-231 and SKBR3 cells. The total RNA was extracted from the mammospheres from breast cancer cells. The markers such as CD44, CD24, and ALDH1 were assayed by real-time RT-PCR. **G** Extracellular cellular acid rate (ECAR) in cancer with CAFs-sEV or NFs-sEV treatment. ECAR was measured by the Seahorse XF Cell Mito Stress Test in MDA-MB-231 cells. Measurements were performed in triplicates. **H** Glycolysis and glycolytic capacity analysis from (**G**). **I** ECAR in cancer with CAFs-sEV or NFs-sEV treatment. ECAR was measured by the Seahorse XF Cell Mito Stress Test in SKBR3 cells. Measurements were performed in triplicates. **J** Glycolysis and glycolytic capacity analysis from (**I**). **K**, **L** The expression of stem cell markers in breast cancer cells. MDA-MB-231 and SKBR3 cells were treated with CAFs-sEV or NFs-sEV for 48 h and total protein was extracted for western blot analysis. *p < 0.05, **p < 0.01.

To answer whether CAFs derived sEVs play roles in the breast cancer stem cell properties, MDA-MB-231 and SKBR3 cells were co-cultured with CAFs-sEV or NFs-sEV and stem cell formation was evaluated using sphere formation assay. It was verified that breast cancer cells could uptake the sEVs from CAFs or NFs (Fig. 2A). After that, MDA-MB-231 and SKBR3 cells were treated with CAFs-sEV or NFs-sEV for 48 h, sphere formation was assayed and the results indicated that CAFs-sEV promoted breast cancer cells showing more sphere numbers (Fig. 2B–D). The stem cell markers including CD44, SOX2, and ALDH1 were enhanced on transcriptional levels (Fig. 2E, F). Glycolysis is a character of cancer cells. To know whether CAFs-sEV influenced cancer cell glycolysis, ECAR was evaluated and CAFs-sEV significantly increases cell ECAR in MDA-MB-231 (Fig. 2G, H) and SKBR3 cells (Fig. 2I, J). The data from western blotting analysis also verified that CD44, SOX2, HK2, GLUT1, and ALDH1 protein levels increased in the cells with CAFs sEVs treatment in MDA-MB-231 cells (Fig. 2K) and SKBR3 cells (Fig. 2L). These data indicated that sEVs from the CAFs increased the population of cancer stem cells and glycolysis.

### MiRNA profile in the sEVs from CAFs and NFs

**Fig. 3:**
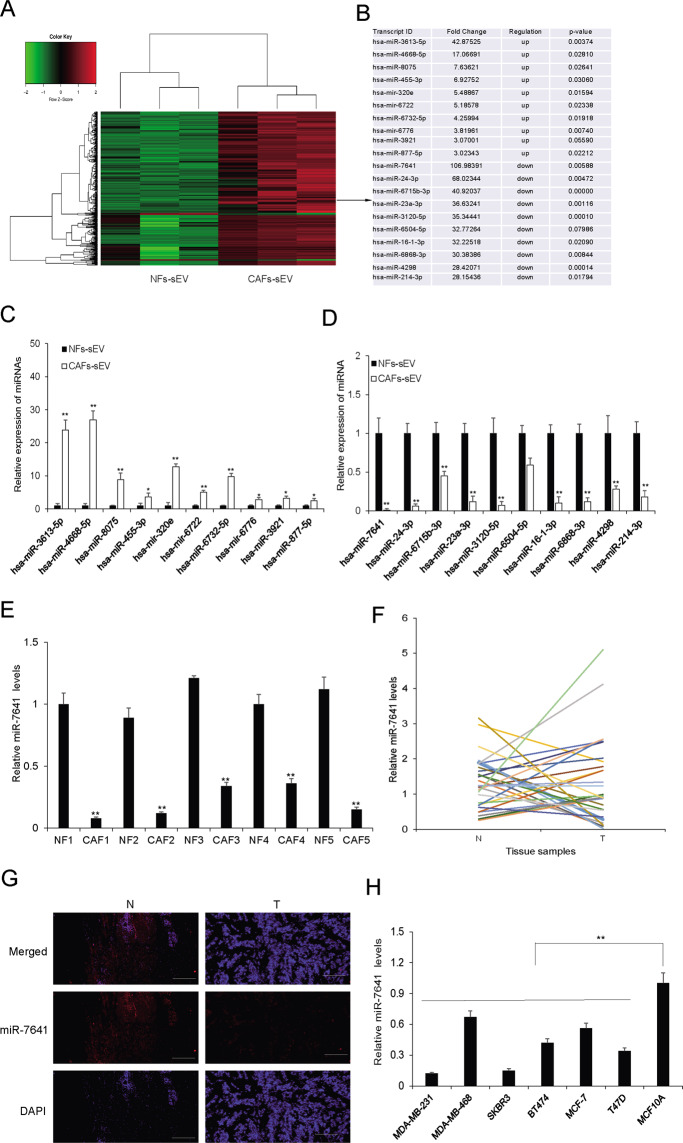
MiRNA profile in the sEVs from CAF and NF. **A** The heatmap of miRNA array assay. **B** The top ten up-regulated and down-regulated miRNAs were listed. **C**, **D** The top ten up-regulated or down-regulated exosomal miRNAs verification in CAFs sEVs. miRNA levels were evaluated by real-time RT-PCR. **E** MiR-7641 expression in five paired sEVs from CAFs and NFs of breast cancer tissues. **F** MiR-7641 expression in breast cancer tissues and their adjacent tissues (*n* = 79). N: adjacent normal tissues; T: breast cancer tissues. **G** MiR-7641 expression in breast cancer tissues and their adjacent tissues using FISH assay. N: adjacent normal tissues; T: breast cancer tissues. **H** The expression of miR-7641 in breast cancer cells with different subtypes and normal breast epithelial cells (MCF10A). *p < 0.05, **p < 0.01.

To further investigate the molecular mechanism in regulating CAFs sEVs on breast cancer cells, sEVs from CAFs and NFs were isolated and total RNA was prepared for miRNA array assay. There were 14 up-regulated miRNA and 530 down-regulated miRNAs in CAFs derived sEVs compared with NFs-sEVs (Fig. 3A) and the top up-regulated and down-regulated miRNAs were listed (Fig. 3B). qRT-PCR was used to verify the expression of the miRNAs (Fig. 3D, E). MiR-7641 was significantly down-regulated in CAFs-sEV (Fig. 3E) and chosen for further investigation. Its expression was confirmed in more breast cancer tissues (Fig. 3F, G). It was also evaluated in breast cancer cells (Fig. 3H). It was also verified that miR-7641 levels were lower in the cells with CAFs-sEV treatment than cells with NFs-sEV treatment (supplementary Fig. S1). These data indicated that miR-7641 was lower in CAFs, breast cancer tissues, and cells than their controls.

### MiR-7641 regulated cancer stem cell properties and glycolysis

**Fig. 4:**
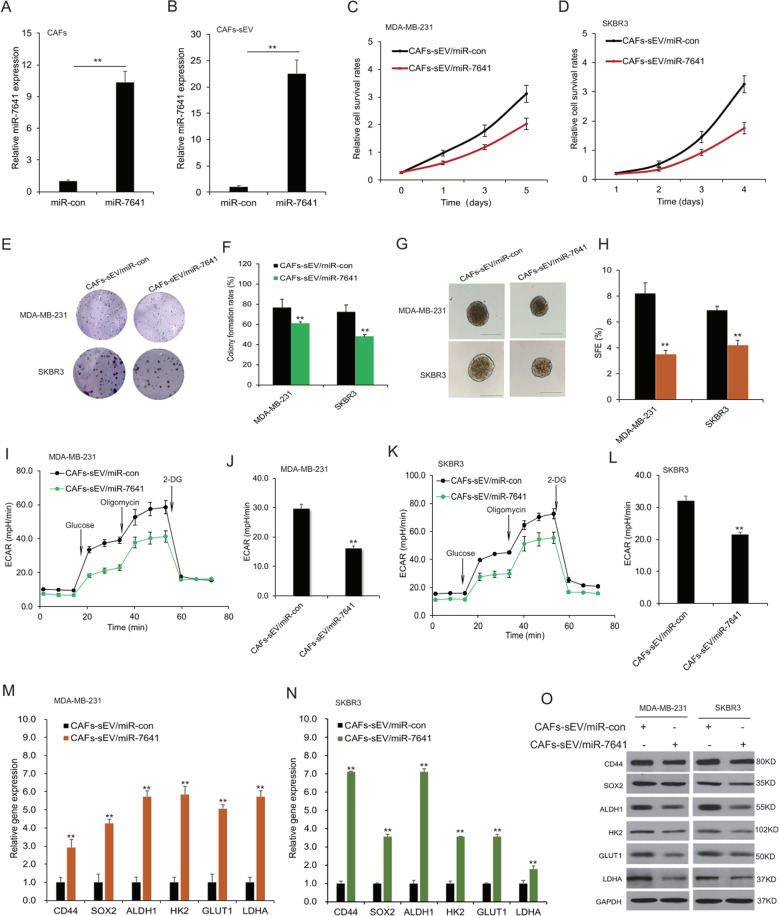
MiR-7641 regulated cancer stem cell properties and glycolysis. **A** miR-7641 level in CAFs. The total RNA was extracted from CAFs transfected with miR-7641 and its expression was assayed by real-time RT-PCR. **B** miR-7641 levels in sEVs from CAFs. The total RNA was extracted from sEVs of CAFs transfected with miR-7641 (CAFs-sEV/miR-7641) or its control (CAFs-sEV/miR-con) and its expression was assayed by real-time RT-PCR. **C**, **D** miR-7641 suppressed breast cancer cell proliferation. MDA-MB-231 and SKBR3 cells were treated with CAFs-sEV/miR-con or CAFs-sEV/miR-7641 for CCK8 assay in five days. **E**, **F** Colony formation of MDA-MB-231 and SKBR3 cells. MDA-MB-231 and SKBR3 cells were treated with CAFs-sEV/miR-con or CAFs-sEV/miR-7641, and then seeded in 6-well plates, and colonies were observed and taken photos after 10 days. **G**, **H** Sphere formation rates of MDA-MB-231 and SKBR3 cells. MDA-MB-231 and SKBR3 cells were treated with CAFs-sEV/miR-con or CAFs-sEV/miR-7641, and then seeded in 6-well plates, and spheres were observed and taken photos after 7 days. **I**, **G** ECAR in MDA-MB-231 cells. Cells were treated with CAFs-sEV/miR-con or CAFs-sEV/miR-7641, and ECAR was analyzed using seahorse. **K**, **I** ECAR in SKBR3 cells. Cells were treated with CAFs-sEV or CAFs-sEV with miR-7641, and ECAR was analyzed using seahorse. **M**, **N** The expression of stem cell and glycolysis associated genes in breast cancer cells. The total RNA was extracted from breast cancer cells treated CAFs-sEV/miR-con or CAFs-sEV/miR-7641. The markers such as CD44, CD24, and ALDH1 were assayed by real-time RT-PCR. **O** The expression of stem cell and glycolysis associated genes in breast cancer cells. MDA-MB-231 and SKBR3 cells were treated with CAFs-sEV/miR-con or CAFs-sEV/miR-7641 for 48 h and total protein was extracted for western blotting analysis. con: control. *p < 0.05, **p < 0.01.

To know the role of CAFs exosomal miR-7641 in breast cancer cells, CAFs were overexpressed miR-7641 for evaluating cellular behaviors and miR-7641 was upregulated in CAFs (Fig. 4A) and CAFs sEVs (Fig. 4B). The sEVs from CAFs with miR-7641 were used to treat MDA-MB-231 and SKBR3 cells, it was shown that CAFs sEVs with miR-7641 overexpression could suppress cell survival ability by CCK8 array (Fig. 4C, D) and reduced the colony numbers by cell colony formation assay (Fig. 4E, F). Sphere formation assay results showed that CAFs sEVs with miR-7641 overexpression reduced the sphere numbers (Fig. 4G, H). Seahorse EACR data showed that CAFs sEVs with miR-7641 overexpression could decrease EACR of MDA-MB-231 cells (Fig. 4I), and glycolysis was suppressed (Fig. 4J). miR-7641 also decreased EACR in SKBR3 cells (Fig. 4K, L). Consistently, the mRNA levels of CD44, SOX2, ALDH1, HK2, GLUT1, and LDHA decreased in MDA-MB-231 and SKBR3 cells with overexpression of miR-7641 in CAFs-sEV (Fig. 4M, N). The protein levels of them also decreased in the MDA-MB-231 and SKBR3 cells with CAFs-sEV miR-7641 treatment (Fig. 4O). Exosomal miR-7641 from CAFs could suppress cancer cell migration and invasion and it was also verified that when the breast cancer cells were transfected with miR-7641, cell migration and invasion were inhibited (Supplementary Fig. S2). It was also verified that when the breast cancer cells transfected miR-7641 mimics showed lower survival ability, stem cell formation, and glycolysis (Supplementary Fig. S3). The results indicated that the introduction of miR-7641 into CAFs sEVs involved in suppressing breast cancer stem cells properties and glycolysis.

### MiR-7641 regulated HIF-1α expression in breast cancer cells

**Fig. 5:**
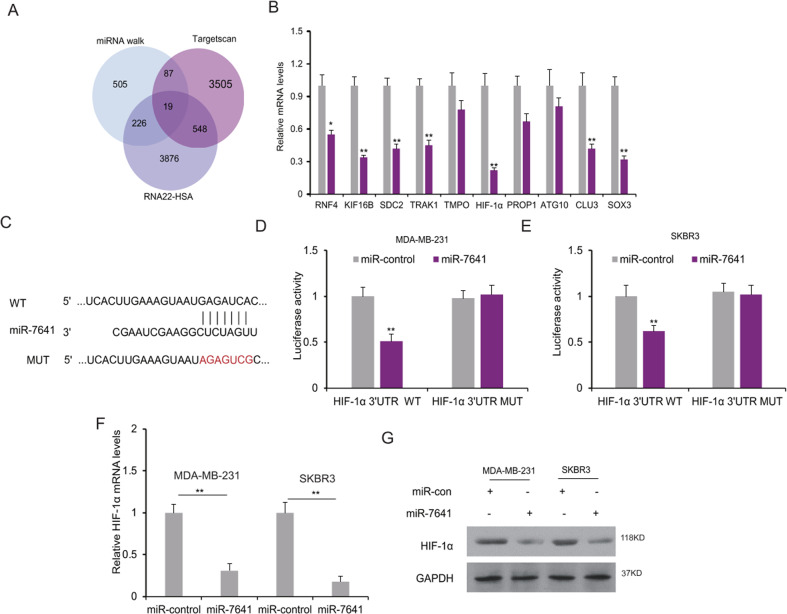
MiR-7641 regulated HIF-1α expression in breast cancer cells. **A** The target gene of miR-7641 was predicted using miRNA walk, Targetcan, and RNA22-HAS. **B** miR-7641 suppressed target gene expression in breast cancer cells. The expression of the possible target genes of miR-7641 was analyzed by real-time RT-PCR. **C** The predicted target sequence of HIF-1α and the mutated sequence of HIF-1α. **D**, **E** MiR-7641 suppressed the luciferase activity of HIF-1α in MDA-MB-231 and SKBR3 cells. Cells were transfected with miR-7641 mimics and plasmids with luciferase reporters and the luciferase activity was assayed by a dual-luciferase reporter system. **F** miR-7641 suppressed HIF-1α mRNA levels in breast cancer cells. Cells were transfected with miR-7641 mimics for 48 h, and total RNA was isolated for real-time RT-PCR analysis. **G** HIF-1α protein levels in breast cancer cells with miR-7641 overexpression. Cells were transfected with miR-7641 mimics for 48 h, and total protein was isolated for western blotting. *p < 0.05, **p < 0.01.

To know the molecular mechanism of miR-7641 in breast cancer, the target gene of miR-7641 was predicted and the predicted target genes were chosen to verify and the expression of them was shown in breast cancer cell lines with miRNA inhibition (Fig. 5A). From the primary screening, HIF-1α was one of the most possible target genes of miR-7641 (Fig. 5B). To verify whether HIF-1α is regulated by miR-7641, HIF-1α 3′UTR luciferase reporter vector (Fig. 5C) was introduced into the SKBR3 and MDA-MB-231 cells, and it was found that miR-7641 suppressed the luciferase activity in the two cell lines (Fig. 5D, E). When the cells were transfected with miR-7641, HIF-1α mRNA was suppressed in the cells (Fig. 5F). HIF-1α protein levels were also reduced in the cancer cells with miR-7641 overexpression (Fig. 5G). It was also verified that HIF-1α mRNA and protein levels were down-regulated in the cells with CAFs-sEV/miR-7641 treatment (Supplementary Fig. S4). The data indicated that HIF-1α was a direct downstream gene of miR-7641 in breast cancer cells.

### MiR-7641 regulated breast cancer cell stemness and glycolysis via HIF-1α

**Fig. 6:**
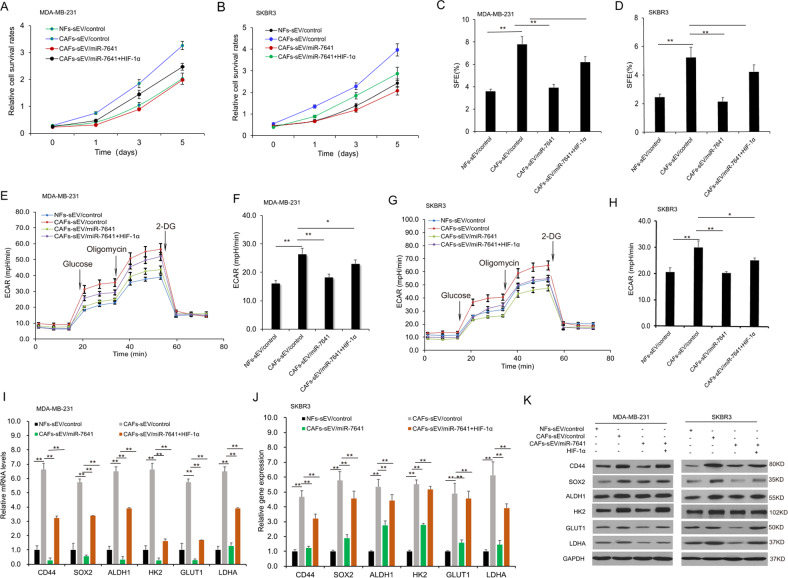
MiR-7641 regulated cancer cell stemness and glycolysis via HIF-1α. **A**, **B** Cell proliferation analysis of SKBR3 and MDA-MB-231 cells. Cells were overexpressed HIF-1α and cultured with sEVs from NFs, CAFs, or CAFs with miR-7641 overexpression. Cell survival ability was assayed by the CCK8 kit. **C**, **D** Sphere formation analysis of spheres in SKBR3 and MDA-MB-231 cells. Cells were overexpressed HIF-1α and cultured with sEVs from NFs, CAFs, or CAFs with miR-7641 overexpression. The photos were taken under a microscope. **E**, **F** ECAR in MDA-MB-231 cells. Cells were overexpressed HIF-1α and cultured with sEVs from NFs, CAFs, or CAFs with miR-7641 overexpression, and ECAR was analyzed using seahorse. **G**, **H** ECAR in SKBR3 cells. Cells were overexpressed HIF-1α and cultured with sEVs from NFs, CAFs, or CAFs with miR-7641 overexpression, and ECAR was analyzed using seahorse. **I**, **J** The expression of stem cell and glycolysis markers in SKBR3 and MDA-MB-231 cells. Cells were overexpressed HIF-1α and cultured with sEVs from NFs, CAFs, or CAFs with miR-7641 overexpression. Stem cell and glycolysis-related genes were assayed by real-time RT-PCR. **K** Western blot analysis of the expression of cancer stemness and glycolysis associated proteins in MDA-MB-231 and SKBR3 cells. Cells were overexpressed HIF-1α and cultured with sEVs from NFs, CAFs, or CAFs with miR-7641 overexpression, and protein was isolated for western blotting. *p < 0.05, **p < 0.01.

To know the role of miR-7641 in breast cancer stemness and glycolysis by HIF-1α, breast cancer cells were in the presence of the sEVs with miR-7641 or HIF-1α overexpression, and cell survival ability was assayed by CCK8 and it was found that cell survival ability was suppressed in the cells with CAFs/miR-7641 sEVs and HIF-1α could recue the progression (Fig. 6A, B). HIF-1α levels increased significantly in breast cancer cells with lentivirus-mediated HIF-1α overexpression (Supplementary Fig. S5). The sphere formation assay verified that stemness was suppressed in the cells with CAFs/miR-7641 sEVs and HIF-1α could recue it (Fig. 6C, D). To know the role of miR-7641 in breast cancer cell glycolysis, MDA-MB-231 and SKBR3 cells were exposed to sEVs from CAFs. Cells with CAFs-sEV treatment showed higher levels of ECAR and miR-7641 could reduce ECAR in MDA-MB-231 cells (Fig. 6E, F) and SKBR3 cells (Fig. 6G, H). The stem cell and glycolysis markers including CD44, SOX2, ALDH1, GLUT1, HK2, LDHA suppressed in the presence of CAFs-sEV with miR-7641, and HIF-1α could recue the expression (Fig. 6I, J) using real-time RT-PCR and western blotting (Fig. 6K). The results indicated that miR-7641 involved in regulating breast cancer stemness and glycolysis by the HIF-1α pathway.

## Discussion

Signals promoting tumorigenesis are from tumor stroma and their microenvironment. CAFs, the main component of tumor stroma, show remarkable capacities to enhance tumor initiation. The sEVs secret miRNAs which transfer from CAFs to cancer cells [16]. Since miRNAs are important regulators in the cancer microenvironment [17, 18], their roles and mechanism in the tumor stroma are still unclear. Here, the study demonstrated that the low levels of exosomal miR-7641 in CAFs supported breast cancer cells showing cancer stem cell properties and glycolysis.

From the miRNA assay analysis, miRNA expression profiles from CAFs and NFs were performed and it was found that there was a small group of differentially expressed miRNAs in the sEVs of CAFs and NFs. Our expression profile identified a set of miRNAs correlated with cell motility and ECM organization. MiR-7641 is reported as a tumor suppressor in cancer progression [18, 19]. MiR-7641 is reported to regulate ribosomal proteins and might be a potential candidate target for cancer therapy [18]. miR-7641 induces endothelial differentiation from human embryonic stem cells by regulating CXCL1 expression [19]. Our study demonstrated that miR-7641 was lower in CAFs than it in NFs.

CAFs or CAFs-derived sEVs promoted breast cancer cell stemness [20, 21]. The study for the first time demonstrated that miR-7641 suppressed breast cancer cell stemness by HIF-1α. The cellular functions like stemness were performed that it was demonstrated that sEVs from CAFs with miR-7641 suppressed breast cancer sphere formation ability. The study suggested that miR-7641 involved in the progress of cancer stem cells transforming cancer cells. One of a mechanism of miR-7641 in suppressing cancer stemness might down-regulate HIF-1α expression in breast cancer cells.

Cell metabolism altering is a hallmark of cancer, which is one of the canonical tumor cell traits. Glycolysis (also known as the Warburg effect) is considered a critical phenotype. Glycolysis is a metabolic pathway that produces two molecules each of pyruvate and ATP from a glucose molecule through sequential and oxygen-independent enzymatic reactions. The sEVs secreted from CAFs can significantly reprogram the metabolic machinery, which regulates cancer cell metabolism [22–24]. HIF-1α is ubiquitously expressed at low, basal levels in all tissues in healthy individuals in normoxia. HIF-1α expression is tightly modulated by different pathways, enzymes, and non-coding RNAs [25–30]. HIF-1α involves various cellular physical processes such as proliferation, drug resistance, glycolysis and etc. We found that CAFs-derived sEVs increased breast cancer cell glycolysis. Further investigation showed that CAFs-derived sEVs with low levels of miR-7641 promotes breast cancer cell glycolysis and HIF-1α was regulated by miR-7641. Previous studies showed that HIF-1α involved in cancer cell metabolism by regulating HK2, GLUT1, and others [25–30]. The study demonstrated that miR-7641 decreased breast cancer cell glycolysis by HK2, GLUT1, LDHA which regulated by HIF-1α.

**Fig. 7:**
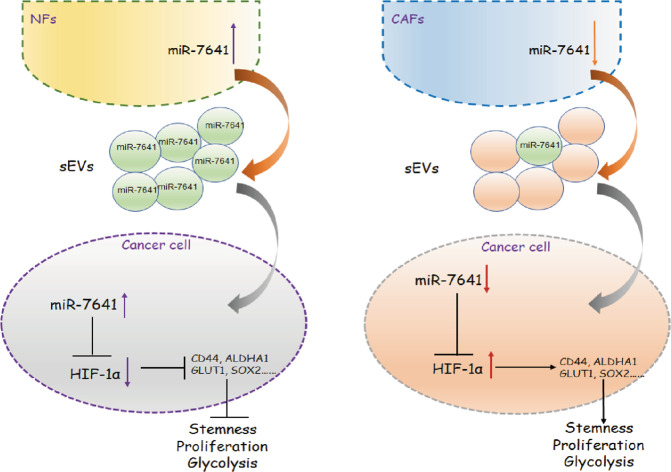
A schematic diagram of the mechanism. CAFs derived sEVs with low miR-7641 promoted breast cancer cell proliferation, stem cell properties, and glycolysis via HIF-1α.

All the factors in tumor stroma may contribute to forming a stem cell or pre-metastatic microenvironment, which accelerates tumor growth. The findings showed that CAFs sEVs involved in cancer cell stemness and glycolysis in the tumor microenvironment. In the presence of CAFs derived sEVs with low miR-7641 levels, breast cancer cells showed more survival ability, more stem cells, and glycolysis by up-regulating HIF-1α expression; however, in the presence of NFs derived sEVs with higher levels of miR-7641, breast cancer cell proliferation, stem cell properties, and glycolysis were suppressed via down-regulating HIF-1α expression (Fig. 7). The study suggested that introduction of miR-7641 in breast cancer cells could be a new way to treat breast cancer.

## Conclusions

The findings showed that CAFs-sEV involved in cancer stem cell phenotype in the tumor microenvironment. It was demonstrated that exosome miR-7641 was an important player in the communication between breast cancer cells and the cellular component of the tumor niche suppressed the breast cancer stem cell population and glycolysis by targeting HIF-1α. We hope the findings will benefit future cancer therapy by targeting the tumor microenvironment.

## Materials and methods

### Cell culture

The cancer cell lines including SKBR3, T47D, MDA-MB-468, MDA-MB-231, MCF-7, and MCF10A were stored in our lab which originally was from American Type Culture Collection (ATCC, Manassas, VA, USA). SKBR3, T47D, and MCF-7 cells were cultured in DMEM/F12 (Hyclone) containing 10% fetal bovine serum (FBS) and 1% penicillin–streptomycin. MDA-MB-468 and MDA-MB-231 cells were maintained in L15 medium with 10% FBS and 1% penicillin–streptomycin. MCF10A cells were cultured in DMEM/F12 containing 5% horse serum, 20 ng/ml EGF and 10 ng/ml insulin, and 1% penicillin–streptomycin. The cells were cultured in a chamber with 5% CO_2_ at 37 °C.

### Breast cancer samples

Breast cancer tissues and their adjacent non-tumor tissues were acquired from Zhongshan Hospital, Fudan University (Shanghai, China). The studies involving human participants were reviewed and approved by the ethics committee. The patients provided written informed consent to participate in this study. All the tissues were stored at −80 °C for use.

### Primary fibroblasts isolation from breast cancer fresh tissues

The fresh tissues from the breast cancer patients were collected in 10 ml phosphate-buffered saline (PBS) supplemented with 2% (double-strength) penicillin–streptomycin and 0.1% (0.25 μg/mL) amphotericin B (Fungizone; Thermo Fisher Scientific, Waltham, USA) and washed three times with PBS. Tissues were cut into 2–3 mm^3^ fragments in sterile conditions, digested using collagenase I (1 mg/ml) overnight, washed with PBS, and then the cells were cultured in 6-well plates containing DMEM/F12 (Hyclone, USA) supplemented with 20% FBS, 2% penicillin–streptomycin for 24 h. The non-attached cells were removed and the attached cells were cultured. The growth medium was changed every 3–4 days. The attached cells were verified as fibroblasts, which were regularly expanded in the standard manner.

### sEV purification

Cells were seeded in 10 cm plates in media without FBS. After 48 h, supernatant fractions were collected from the cells. The supernatant was centrifugated at 500 g for 10 min and then 20,000 g for 20 min. PBS was used to resuspend the exosome pellet, which was ultra-centrifugated at 100,000 g for 70 min. The sEVs were stored at −80 °C for further experiments.

### TEM

sEVs from NFs or CAFs were extracted. The sEVs were re-suspended with low salt PBS and dropped onto 100-mesh copper grid. The sEVs were negatively stained by 2% phosphotungstic acid for 3 min, dried for 15–20 min. The sEVs were observed under a TEM (FEI Tecnai G2 Spirit, Thermo Fisher Scientific) at 80 kV.

### sEVs labeling and transferring

sEVs isolated from CAFs were labeled and incubated with DiO (Thermo Scientific, USA) (10 μM) for 20 min at 37 °C. The labeled sEVs were washed in PBS, and then centrifuged at 100,000×*g* for 70 min at 4 °C. MDA-MB-231 cells (3 × 10^4^ cells per well) grown on coverslips were incubated with DiO-labeled sEVs (25 µg/ml) for 24 h [12]. To identify the transfer of sEVs derived miR-7641, Cy3-labeled miR-7641 was introduced into CAFs. The Cy3-miR-7641-expressing CAFs were then co-cultured with MDA-MB-231 cells for 48 h using a transwell chamber. The cells were then prepared for immunofluorescence as described above and the internalized sEVs miR-7641 was measured by a confocal microscope.

### Western blot analysis

Cells or sEVs were lysed in a lysis buffer and centrifugated by 4000 g for 30 min at 4 °C. BCA method was used to detect the protein concentration. The protein was separated by SDS-PAGE and transferred onto PVDF (GE life). The PVDF with protein was probed with primary antibodies CD81, CD63, GM130, CD44, SOX2, ALDH1, GLUT1, HK2, LDHA, and HIF-1α with 1:1000 and the secondary antibodies (CST, USA) with 1:2000. Then, the PVDF was stained with horseradish peroxidase-labeled secondary antibodies. The protein was visualized using an enhanced chemiluminescence detection kit (Absin, China).

### MiRNA array

There were three pairs of NFs and CAFs that were mixed. MiRNA array was performed using the Affymetrix miRNA array platform. FlashTag Biotin HSR RNA Labeling Kit (Affymetrix P/N 901910, Thermo Scientific, USA) was used to label the miRNAs. The Affymetrix miRNA 3.0 array was scanned by the Affymetrix Scanner 3000 (Affymetrix, Thermo Scientific, USA). Affymetrix GeneChip command console software (version 4.0, Affymetrix) was used to analyze the data.

### Real-time RT-PCR

Total RNA from breast cancer cells with transfection or tissues was extracted using EZ total RNA isolation kit according to the manufacturer’s protocol. All the primers were ordered from Ruibo (Shanghai, China). After reverse transcription, PCR was run on the 7500 Real-Time PCR system. The program of PCR was 95 °C for 10 min, 40 cycles at 95 °C for 10 s, 60 °C for 30 s, 72 °C for 10 s, followed by a melting curve analysis. GAPDH or U6 snRNA was used as the internal controls. The results were analyzed by 2^−ΔΔCt^ method.

### Bioinformatic analysis

The putative target genes were predicted using Targetscan (TargetScanHuman 7.2), miRNA walk (http://mirwalk.umm.uni-heidelberg.de/), and RNA22-HAS (https://cm.jefferson.edu/data-tools-downloads/rna22-full-sets-of-predictions/).

### Oligonucleotide transfection, vector construction, and stable transfection

Cells were transfected with small interfering RNAs (siRNAs), miRNAs mimics, or miRNA inhibitors (GenePharma, China) using Lipofectamine 2000 (Invitrogen, USA). The overexpression of HIF-1α was ordered from Genechem (Shanghai, China). The lentiviral particles with miR-7641 inhibitors or miR-7641 overexpression were prepared in 293T cells. HEK-293T cells were transfected with overexpression vectors, psPAX2, and PMD2.G using Lipofectamine 2000. Lentiviruses were harvested and filtered. Cells were infected with a virus and selected with puromycin. The expression of miR-7641 or HIF-1α in breast cancer cells was evaluated by real-time RT-PCR.

### Cell proliferation assay

Breast cancer cells were treated with sEVs from CAFs with miR-7641 overexpression, miR-7641 down-regulation and then seeded in a 96-well plate. Cell proliferation was evaluated by CCK8 assay. The survival rates were analyzed. For colony formation, the cells were seeded in 6-well plates for 10–14 days and the colonies with more than 50 cells were counted and analyzed.

### Wound healing assay and cell invasion

Breast cells were treated with sEVs from CAFs with various treatments. The cells were seeded in 12-well plates and made a wound using 200 µl tips. The wounds were taken photos, the widths were measured and the migration ability was analyzed. For cell invasion ability, the cells with sEVs treatment were seeded in the up-chamber of the transwell system. After the cells in the up-chamber were removed, the invaded cells in the membrane were fixed in 100% methanol and then dyed by 0.1% crystal violet. The photos of the invaded cells in three fields were taken and cell invasion ability was analyzed.

### Luciferase analysis

Breast cancer cells were seed in a 24-well plate. The next day, the cells were co-transfected with luciferase reporter, miR-7641 mimics, and Renilla luciferase vector (Genomeditech, China) using Lipofectamine™ 2000 (Invitrogen, USA). 48 h later, the luciferase and Renilla activity of these samples was measured by Dual-Luciferase Reporter Assay kit (Promega, USA).

### Sphere formation assay

SKBR3 and MDA-MB-231 cells (5000 cells/well) were cultured in a 6 well ultralow attachment plate (Corning Inc., USA). These cells were cultured for 7–10 days in a stem cell medium with 20 ng/ml EGF and 20 ng/ml bFGF and 1 × B27 (Thermo Scientific, USA, IL) at 37 °C under 5% CO_2_. The mammospheres with a diameter larger than 50μm were counted under inverted microscopy or harvested by centrifugation for other experiments.

### ECAR measurement

Assays were performed using the Seahorse XFe96 analyzer (Seahorse Bioscience, Agilent) according to the manufacturer’s instructions. For the assay, cells were plated in XFe96 Cell Culture Microplates (Agilent Technologies) at a cellular density of 30,000 cells/well and 20,000 cells/well with MDA-MB-231 and SKBR3 cells respectively. ECAR was measured with an XF96 analyzer in XF base medium (pH 7.4) containing 1 mM glutamine following sequential additions of glucose (10 mM), oligomycin (1 mM), and 2-DG (50 mM). Data were analyzed by the Seahorse XF Glycolysis Stress Test Report Generator package.

### RNA FISH

Cy3-labeled oligonucleotide probe for miR-7641 was applied for RNA FISH. The paraffin section of breast cancer samples was deparaffinized with 100% xylene and rehydrated with different graded ethanol. For RNA FISH of co-localization of miR-7641, cells were seeded in a glass-bottom dish. Then they were incubated with prehybridization solution at 37 °C for 30 min and the probes (Ribobio, China, 20 μM) were added to slides or dishes individually and hybridized overnight. Then they were washed with buffer I (4 × SSC, 0.1% Tween-20) three times, wash with buffer II (2 × SSC) for once, and wash with buffer III (1 × SSC) for once. After being washed with PBS, they were incubated with DAPI to stain the cell nuclear.

### Murine xenograft model and Immunohistochemistry (IHC)

Female BALB/c nude mice with five-week-old were used to establish the breast cancer models using MDA-MB-231 cells (1 × 10^7^) with 50 µg sEVs purified from the culture supernatants of NFs or CAFs by subcutaneously injecting into the fat pad. Tumor volume was monitored twice a week and calculated using the formula: volume (mm^3^) = width^2^ × length/2. After 3 weeks following the inoculation, the mice were killed and tumor samples were weighted. Immunohistochemistry to detect Ki67 in breast cancer samples was performed as described previously [10].

### Statistical analysis

Statistical analyses were performed using SPSS 16.0 and Excel. All quantitative experiments were repeated with at least three independent biological repeats and are presented as the means ± SD (standard deviation). Quantitative data were analyzed by either one-way analysis of variance (ANOVA) (multiple groups or parametric generalized linear model with random effects for tumor growth and CCK8 assay) or *t*-test (two groups). *p* < 0.05 was considered statistically significant.

## Supplementary information

Supplementary Figure legends

FigureS1

FigureS2

FigureS3

FigureS4

FigureS5
