# Supplementary material for: Carcinoma associated fibroblasts small extracellular vesicles with low miR-7641 promotes breast cancer stemness and glycolysis by HIF-1α
Source: Cell Death Discov. 2021 Jul 8;7:176. doi: 10.1038/s41420-021-00524-x (PMC8266840; doi:10.1038/s41420-021-00524-x)
Supplement: Supplementary file 1 — Supplementary Figure legends [file 41420_2021_524_MOESM1_ESM.docx]

**Fig. S1** **miR-7641 levels in MDA-MB-231 and SKBR3 cells.** MDA-MB-231 and SKBR3 cells were treated with NFs-sEV or CAFs-sEV for 48h and then the levels of miR-7641 were assessed by real time RT-PCR. ***p*<0.01.

**Fig. S2 MiR-7641 regulated breast cancer cell migration and invasion. A-B** Wound healing assay ­­­­­­was used to examine cell migrati­­­on of MDA-MB-231 and SKBR3 cells with CAFs-sEV/miR-con or CAFs-sEV/miR-7641 treatment (25ug/ml). **C-D** Transwell sys­­­­­tem was used to examine cell invasion of MDA-MB-231 and SKBR3 cells with CAFs-sEV/miR-con or CAFs-sEV/miR-7641 treatment (25ug/ml). Scale bars represented 100um. con: control. ***p*<0.01.

**Fig. S3 MiR-7641 regulated cancer stem cell properties and glycolysis. A-B** miR-7641 suppressed breast cancer cell proliferation. MDA-MB-231 and SKBR3 cells were infected with lentivirus-mediated miR-7641 and then cell proliferation was assayed using CCK8 assay at 1, 3 and 5 day. **C-D** Sphere formation rates of MDA-MB-231 and SKBR3 cells. MDA-MB-231 and SKBR3 cells were treated with were infected with lentivirus-mediated miR-7641, and then seeded in 6-well plates and sphere were observed and taken photos after 7 days. **E-F** ECAR in MDA-MB-231 cells. MDA-MB-231 and SKBR3 cells were infected with lentivirus-mediated miR-7641 and ECAR was analyzed using seahorse. Scale bars represented 100um. ***p*<0.01.

**Fig. S4 HIF-1a expression in breast cancer cells.** **A** MDA-MB-231 and SKBR3 cells were co-cultured with NF-, CAF- or CAF-miR-7641 sEVs and HIF-1α expression was assessed by RT-PCR. **B** MDA-MB-231 and SKBR3 cells were co-cultured with NF-, CAF- or CAF-miR-7641 sEVs and HIF-1α expression was assessed by western blotting. ***p*<0.01.

**Fig. S5 HIF-1α expression in breast cancer cells.** MDA-MB-231 and SKBR3 cells were infected with lentivirus mediated HIF-1α overexpression and HIF-1α expression was assessed by western blotting.
