# Supplementary figures and images for: Carcinoma associated fibroblasts small extracellular vesicles with low miR-7641 promotes breast cancer stemness and glycolysis by HIF-1α
Source: Cell Death Discov. 2021 Jul 8;7:176. doi: 10.1038/s41420-021-00524-x (PMC8266840; doi:10.1038/s41420-021-00524-x)

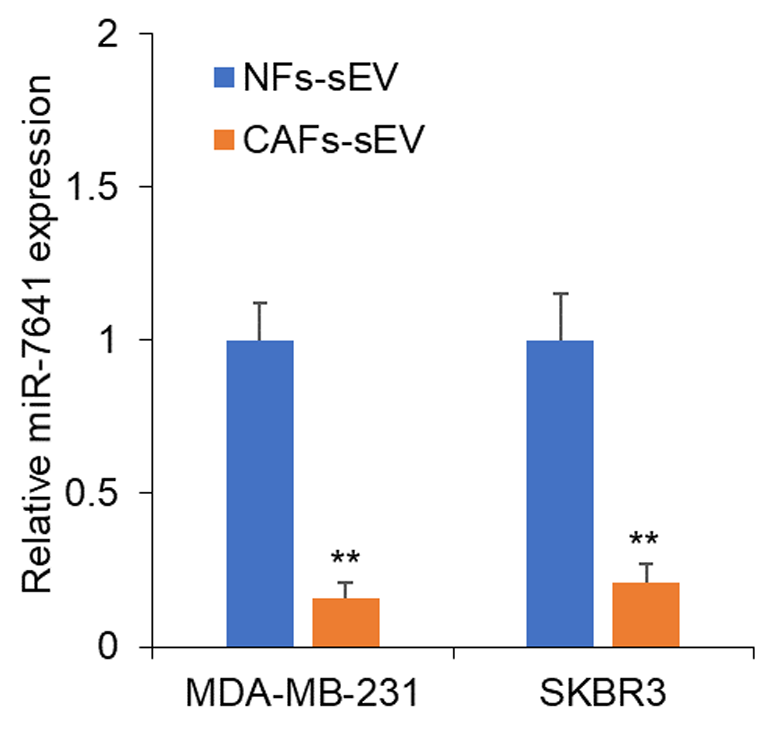

Supplement: Supplementary file 2 — FigureS1 [file 41420_2021_524_MOESM2_ESM.tif]

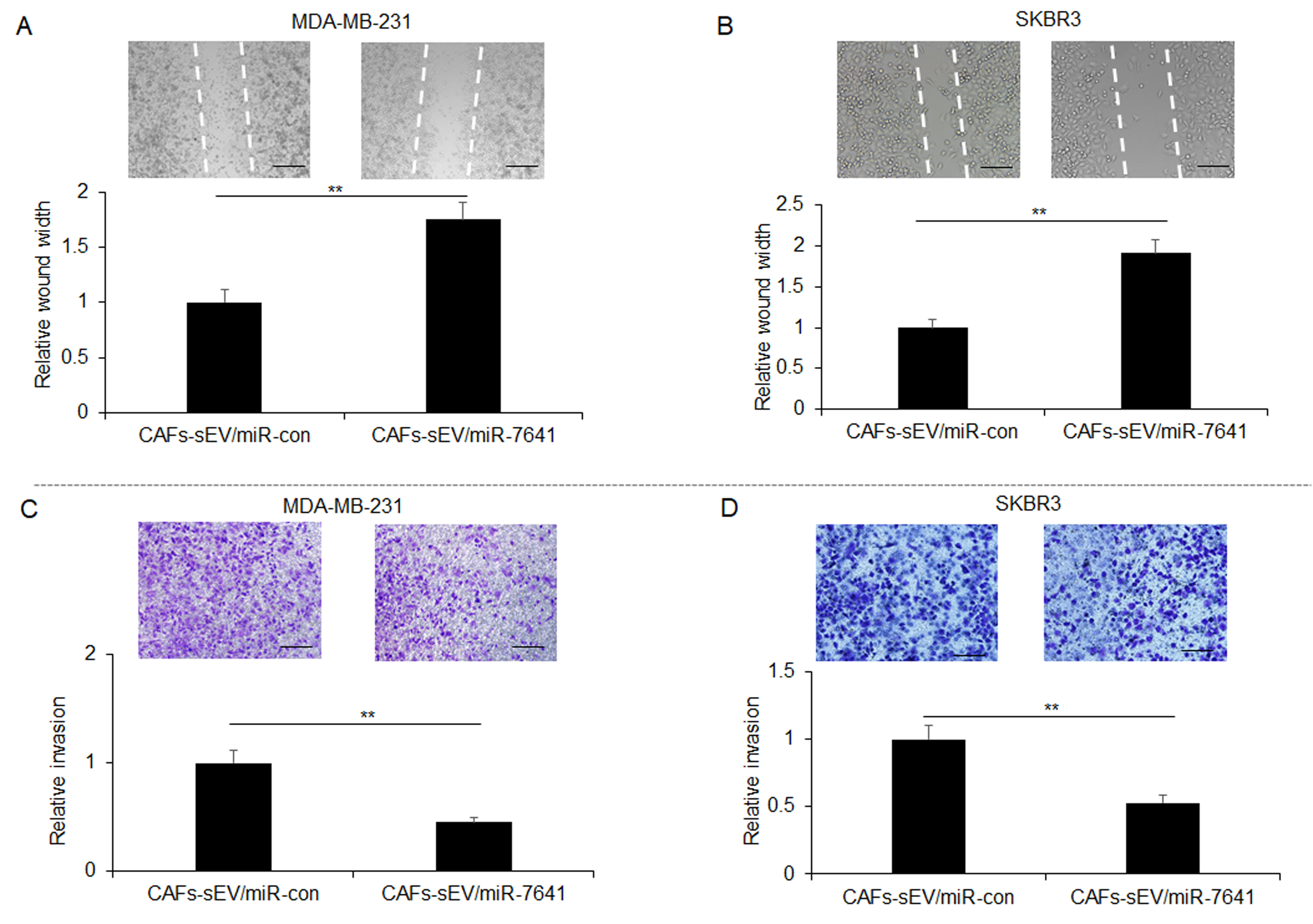

Supplement: Supplementary file 3 — FigureS2 [file 41420_2021_524_MOESM3_ESM.tif]

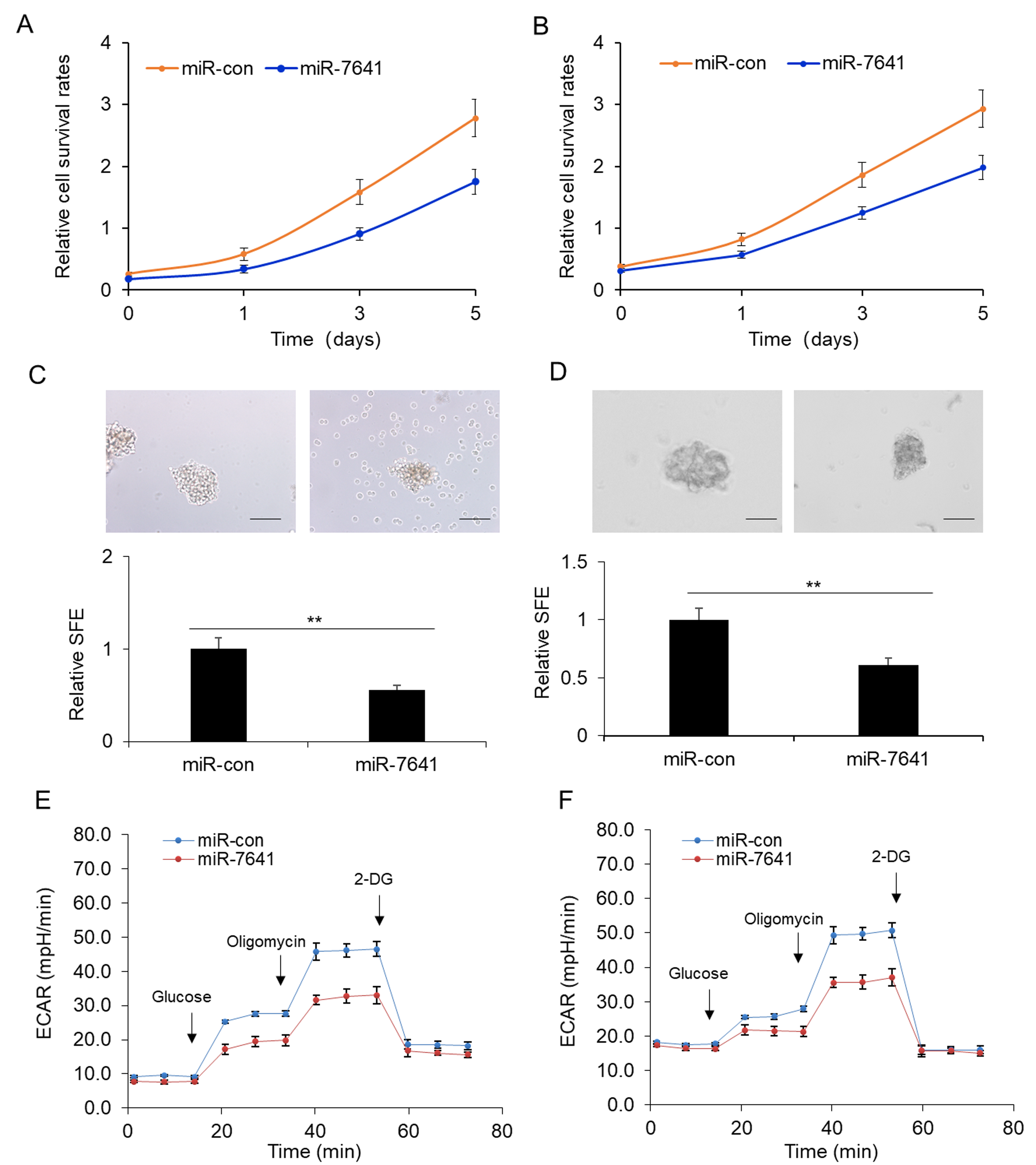

Supplement: Supplementary file 4 — FigureS3 [file 41420_2021_524_MOESM4_ESM.tif]

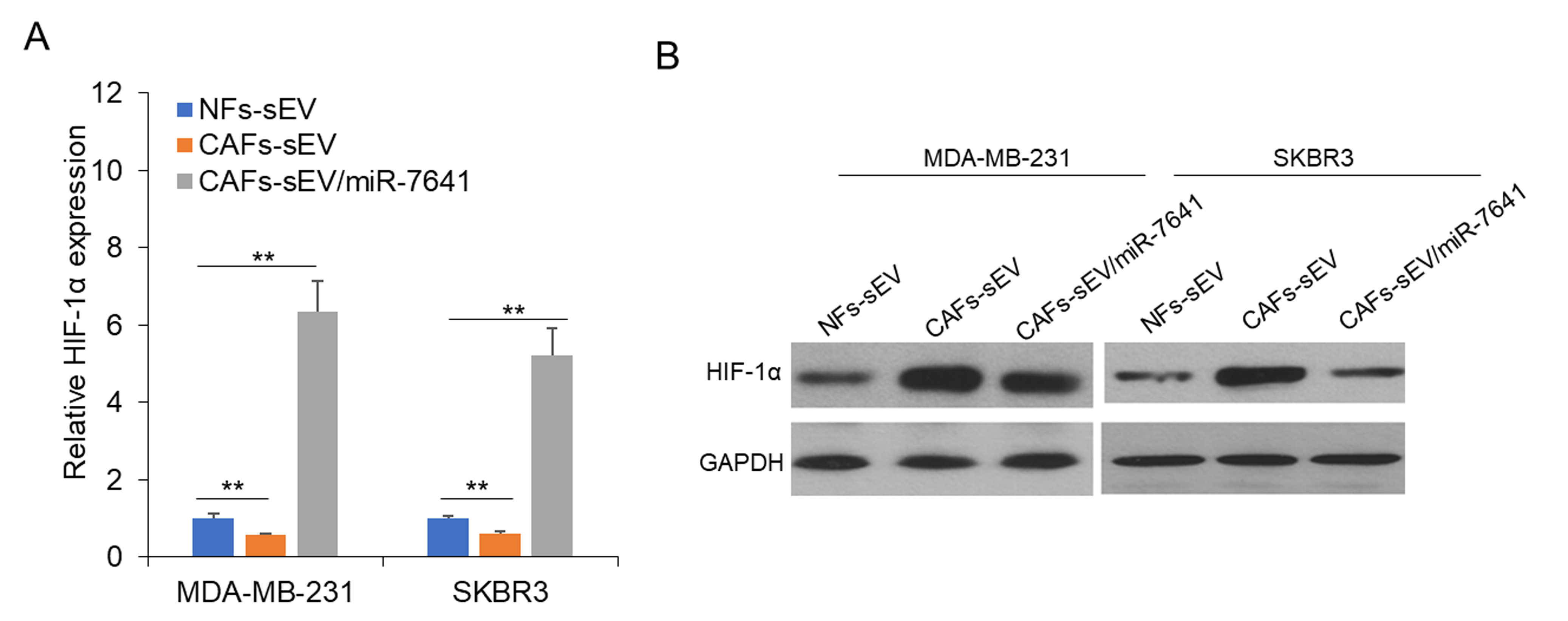

Supplement: Supplementary file 5 — FigureS4 [file 41420_2021_524_MOESM5_ESM.tif]

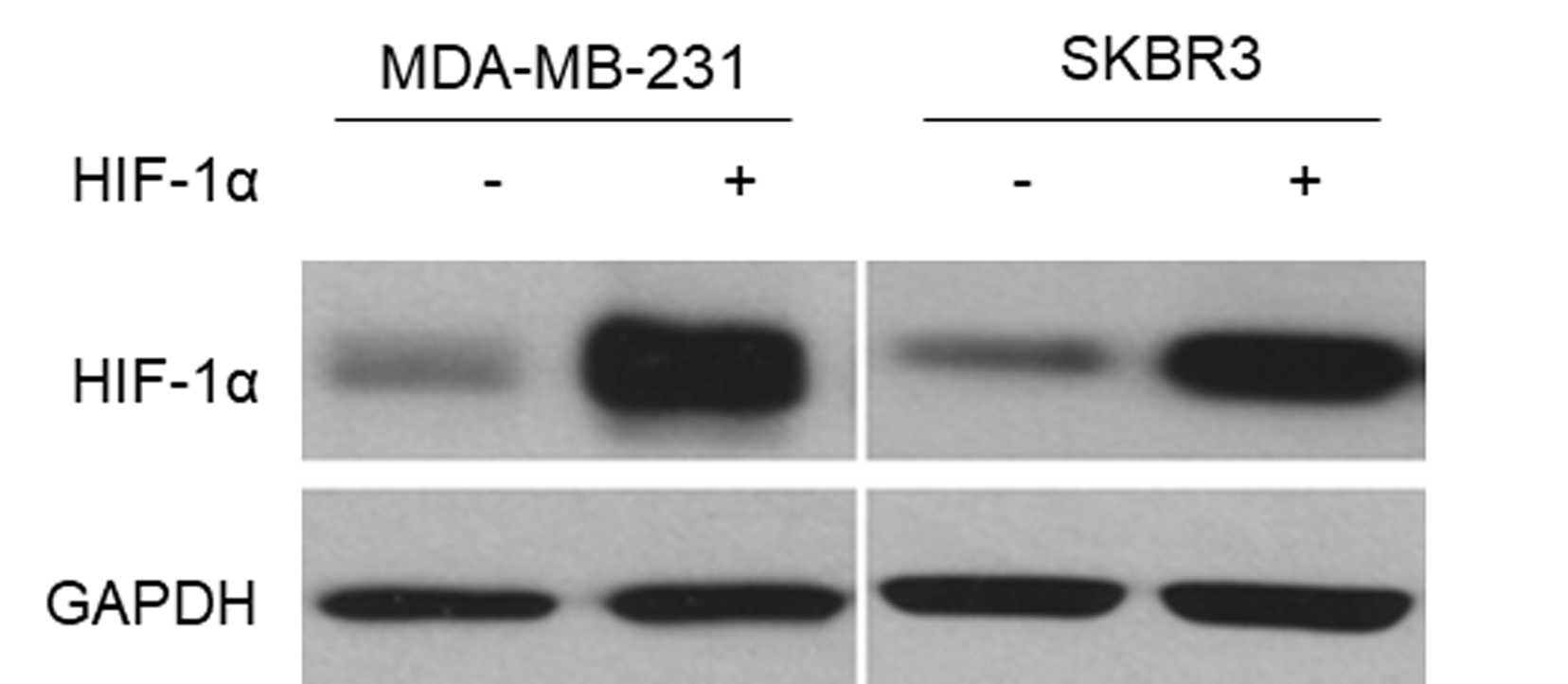

Supplement: Supplementary file 6 — FigureS5 [file 41420_2021_524_MOESM6_ESM.tif]
